# Food Adulteration

**Published:** 1881-06

**Authors:** 


### FOOD ADULTERATION.

Dr. Charles Smart, U. S. A., in the *National Board of Health Bulletin*, January 1st, 1881, submits a report of interest on this subject, prepared by instructions from the National Board of Health. The first adulterated article of food alluded to is tea. Of 109 teas examined, 90 were obtained from sources which should have furnished a pure article, while 19 were obtained from questionable sources. The results of thorough examinations permits the committee to report that they think tea adulteration is not carried on to any very great extent. In England, the chief adulteration of coffee is chicory. Dr. Hassall testified before the Food Commission, in 1855, that of 34 samples which he had examined, 31 contained chicory. Normandy found as much as 75 per cent. of chicory. Roasted wheat, beans, rye and potato flours, mangel-wurzel and a substance resembling acorns, roasted corn, and ground cocoa-ribs, while as adulterations of the chicory were found deal and mahogany sawdust, carrots,

Venetian red and burnt sugar. The reporter pertinently remarks that while in this country we have the credit of making wooden nutmegs, we have never been accused of manufacturing coffee beans from compressed chicory. In England, sand in sugar is a familiar phrase. Most of the sugar there examined was dark in color, mixed with much vegetable extractive, and swarming with sugar-mite. The sugar of the present day is purer. The report concludes that manufacturers of flour do not to any great extent doctor the flour, but that bakers do. Corn meal, bicarbonate of soda, cream of tartar, baking powders, black pepper, pickles, confectionery, and other common household agents, were noticed, and a detail of the method of examining them. It is a useful report.

